# The Effect of Concurrent Plyometric Training Versus Submaximal Aerobic Cycling on Rowing Economy, Peak Power, and Performance in Male High School Rowers

**DOI:** 10.1186/s40798-017-0075-2

**Published:** 2017-02-01

**Authors:** Julian D. Egan-Shuttler, Rohan Edmonds, Cassandra Eddy, Veronica O’Neill, Stephen J. Ives

**Affiliations:** Health and Exercise Sciences Department, Skidmore College, Saratoga Springs, NY USA

**Keywords:** Rowing, Stretch shorten cycle, Oxygen consumption

## Abstract

**Background:**

Plyometric training has been shown to increase muscle power, running economy, and performance in athletes. Despite its use by rowing coaches, it is unknown whether plyometrics might improve rowing economy or performance.

The purpose was to determine if plyometric training, in conjunction with training on the water, would lead to improved rowing economy and performance.

**Methods:**

Eighteen male high school rowers were assigned to perform 4 weeks of either plyometric training (PLYO, *n* = 9) or steady-state cycling below ventilatory threshold (endurance, E, *n* = 9), for 30 min prior to practice on the water (matched for training volume) 3 days per week. Rowing performance was assessed through a 500-m rowing time trial (TT) and peak rowing power (RP), while rowing economy (RE) was assessed by measuring the oxygen cost over four work rates (90, 120, 150, and 180 W).

**Results:**

Rowing economy was improved in both PLYO and E (*p* < 0.05). The 500-m TT performance improved significantly for PLYO (from 99.8 ± 9 s to 94.6 ± 2 s, *p* < 0.05) but not for E (from 98.8 ± 6 s to 98.7 ± 5 s, *p* > 0.05). Finally, RP was moderately higher in the PLYO group post-training (E 569 ± 75 W, PLYO 629 ± 51 W, ES = 0.66)

**Conclusions:**

In a season when the athletes performed no rowing sprint training, 4 weeks of plyometric training improved the 500-m rowing performance and moderately improved peak power. This increase in performance may have been mediated by moderate improvements in rowing power, but not economy, and warrants further investigation.

## Key Points

- Four weeks of plyometric training lead to increased rowing performance, which may be mediated by a moderate increase in peak power.
- Time-matched cycling exercise did not improve performance or peak power; however, rowing economy was improved similarly in both groups.
- These findings suggest that plyometric training is useful for increasing rowing performance.

## Background

Rowing is a high-intensity sport, requiring high strength, power, anaerobic, and aerobic capacity [1–4]. Race times over the typical 2-km rowing race can range from 5.5 to 7 min in elite rowers, indicating a need for a variety of training intensities [1]. It has been estimated that aerobic metabolism contributes 67–84% of the energy requirement during racing [1], and this relative contribution of aerobic and anaerobic energy systems is the same for both on-the-water and ergometer rowing [5]. Because of the relatively high reliance on aerobic metabolism in racing performance [4, 5], endurance training dominates the training programs of most rowers [1]. However, there are multiple determinants of rowing performance [4], indicating that a rower’s training program must encompass aerobic and anaerobic training, as well as training methods to develop peak power such as strength or power training. In fact, Ingham et al. [4] indicated that peak force and power over a maximal five-stroke test were two of the highest correlates to 2-km ergometer performance. As peak power correlates very highly with rowing performance, training for power is a part of many rowers’ training programs [2] which may consist of strength training [6] or heavy rowing [7], performed concurrently with aerobic training. While strength training has been shown to enhance rowing performance [8–10], much less is known about the effect of power training, such as plyometrics, on rowing performance. Additionally, given the minimal equipment required to perform plyometrics versus weight training, plyometrics might have a greater potential for implementation.

There is a variety of ways to improve strength and power, and in many sports, plyometric training is used to increase movement speed and power [11]. Plyometric training is a type of physical conditioning that emphasizes the stretch-shortening cycle and typically involves both open and closed kinetic chain exercises, such as jumping or medicine ball throws [11]. A recent meta-analysis by Sáez-Sáez de Villarreal et al. [12] suggests that plyometric training is an effective method to improve strength and power, even over short periods (<10 weeks). Plyometric training improves power through increased neural drive, changes in muscle coordination, changes in the muscle tendon complex, and changes in muscle size and architecture [11]. It is known that neural adaptations account for strength increases in the early phases of strength training [13, 14]; however, major neural adaptations also occur with plyometric training and lead to the increases in explosive force production [15–17]. Plyometrics have also been shown to increase the cross-sectional areas of both type I and type II muscle fibers [18], without change to the myosin heavy chain ratios [16, 19]. Increased muscle preparatory activity and muscle activation have also been found, leading to plyometrics possibly even preventing injury [20]. However, the application of this training method has traditionally been applied to relatively narrow populations (e.g., football or track and field).

Previously, running economy, or the energy cost of running at a given work rate, has been shown to improve in response to plyometric training [21]. Running economy has even been suggested to be a better correlate of running performance than maximal oxygen consumption (VO_2max_) [22]. Paavolainen et al. [23] were able to show that when endurance runners incorporated plyometric/explosive strength training into their training, 5-km running times and running economy were improved, and all without a change in VO_2max_. This increase in running economy has been suggested to be due to an increase in stiffness of the musculotendinous system, which allows the body to momentarily store and utilize energy absorbed eccentrically by the force of landing [24], but neural mechanisms cannot be excluded as potential contributors [15, 16]. Thus, as plyometrics can improve endurance performance and power, with no chronic detriment when performed concurrently [23], plyometrics has the potential to be an effective means to improve performance in rowers, where the legs contribute significantly to generating stroke power.

To this end, Kramer et al. [3] investigated whether adding 20 min of plyometric training to rowing training would improve rowing performance. After 9 weeks of training, improvement in rowing performance, assessed via a 2.5-km time trial, did not differ between the control and experimental groups [3]. However, there were several limitations to this study: first, the training stimulus or volume of 20 min 3×/week may have been insufficient; second, the 2.5-km time trial (TT) is atypical of rowing; third, training volume was not matched between groups; fourth, the exercise selection was not optimal for rowers; and finally, rowing economy or power were not measured. Interestingly, while the previous study was unable to demonstrate a benefit of plyometrics on rowing performance [3], an estimated 50% of rowing coaches continue to include plyometric training into their training programs to improve rowing power [2]. Coaches that do use plyometrics often perform other strength training methods in addition, as concurrent training has been shown to increase rowing performance [10]. However, it remains to be seen whether plyometrics alone can improve rowing performance and thus validate their use by rowing coaches [7].

Therefore, the purpose of this study was to determine if 4 weeks of plyometric training, versus submaximal aerobic training matched for volume, would increase rowing economy, peak power, or performance in experienced youth rowers when combined with on-the-water training. We hypothesized that rowers who performed plyometric training would improve rowing economy, peak power, and performance over those conducting submaximal steady-state exercise.

## Methods

### Participants

Eighteen male rowers with a minimum of 1 year’s competitive rowing experience were recruited from a local competitive (i.e., multiple state and national championship medals) high school rowing program. The participants had no previous experience with plyometric training. The experimental plyometric (PLYO) and the endurance (E) group each consisted of nine high-school-aged male rowers. The groups were matched with regard to pretraining 500-m times (endurance, E = 98.8 ± 5.8 s; PLYO = 99.8 ± 9.6 s, *p* > 0.05). As a result, there was also no difference in biological age or training age between groups. The physical characteristics of both groups are presented in Table 1. Unfortunately, recent 2000-m rowing performance times for the group were not available, but the team had achieved several state and national medals. All participants, and their legal guardians, provided written informed consent prior to inclusion in this study. This study was approved by the Institutional Review Board of Skidmore College and therefore was performed in accordance with the ethical standards set forth in the Declaration of Helsinki.

**Table 1:** The participant characteristics at baseline and following the 4-week intervention

	Control Group (*n* = 8)		Experimental Group (*n* = 8)	
	Pre	Post	Pre	Post
Age (year)	16 ± 0.6	16 ± 0.6	16 ± 0.8	16 ± 0.8
Stature (cm)	177 ± 4	177 ± 4	179 ± 6	179 ± 6
Mass (kg)	66.5 ± 9.4	66.2 ± 8.6	71.4 ± 6.5	71.8 ± 6.9
Thigh Circumference (cm)	49 ± 4.8	49 ± 3.0	52 ± 4.0	53 ± 3.5
Calf Circumference (cm)	35.4 ± 2.6	34.5 ± 2.5	35.1 ± 2.0	35.2 ± 1.9

Data are presented as mean ± SD

### Intervention

The intervention lasted for 4 weeks, which is defined as a short-term plyometric period [11, 12] and is long enough to elicit significant physiological and/or performance changes [17, 25]. All testing and training was conducted during the fall competition season (September to November). These 4 weeks of training was also selected so that training could be completed before the major competitions for the rowers. Both groups performed 30 min of cross-training before on-the-water practice, 3 days/week, with 48 h in between sessions (e.g., Monday, Wednesday, and Friday). The PLYO group performed 30 min of plyometric exercise, while E performed steady-state cycling. All plyometric sessions contained exercises focusing on vertical explosiveness, such as box jumps, depth jumps, multiple box-to-box jumps, and double leg hops. Furthermore, as the rowing stroke involves opening of the trunk, backwards and overhead throws of medicine balls (10 lbs) were included to train explosive triple extensions. Sessions ranged from 100- to 150-ft contacts in week 1 to 125- to 170-ft contacts in week 4. Medicine ball exercise repetitions were periodized to progressive overload. The volume (number of foot contacts) conforms to recommended guidelines [26]. The full training program can be found in Table 2. A trained member of the research team oversaw every plyometric session and provided feedback on the quality of the movements.

**Table 2:** The plyometric program performed for 30 min, 3 days a week, for 4 weeks

	Week 1	Week 2	Week 3	Week 4
Day 1				
Single leg push-off	3 × 8	3 × 10	3 × 10	3 × 10
Box jump	3 × 5	3 × 6	3 × 6	3 × 6
Depth jump	3 × 5	3 × 5	3 × 5	3 × 5
Squat jump	3 × 15	3 × 20	3 × 20	3 × 20
Backward throw	3 × 6	3 × 6	3 × 6	3 × 6
Overhead throw	3 × 5	3 × 6	3 × 6	3 × 6
Trunk rotation	3 × 5	3 × 6	3 × 6	3 × 6
Foot contacts	100	125	125	125
Day 2				
Double leg hops	3 × 5	4 × 5	4 × 5	4 × 5
5-5-5 squat	3 times	4 times	4 times	4 times
Front cone hops	4 cones, 5 times	4 cones, 5 times	4 cones, 5 times	4 cones, 5 times
Multiple box-to-box jumps	5 times	5 times	5 times	5 times
Wave squat	2 × 5	10 reps	10 reps	10 reps
Box jump	3 × 5	4 × 5	4 × 5	4 × 5
Foot contacts	150	170	170	170
Day 3				
Standing jump over barrier	3 × 5	4 × 5	4 × 5	4 × 5
Lateral jump over barrier	3 × 5	4 × 5	4 × 5	4 × 5
Single leg push-off	3 × 8	3 × 8	3 × 8	3 × 8
Depth jump	3 × 5	4 × 5	4 × 5	4 × 5
Pyramiding box hops	5 rounds	5 rounds	5 rounds	5 rounds
Backward throw	3 × 6	3 × 6	3 × 6	3 × 6
Overhead throw	3 × 5	3 × 5	3 × 5	3 × 5
Foot contacts	120	150	150	150

The endurance group performed 30 min of steady-state cycling at ventilatory threshold at the same time as the experimental group. The “Talk Test” (cycling at an intensity at which a conversation was possible) was used to prescribe cycling intensity and has been shown to be a valid approximation of ventilatory threshold [27]. The athletes were also familiar with this method, as it was used at their club to prescribe low-intensity aerobic workouts. Cycling was used so that one group was not provided with a higher volume of rowing-specific training than the other and is also a common cross-training method for rowers. The endurance group was also supervised. After each group completed the 30 min of training, they proceeded to their on-the-water rowing practice. All subjects completed the same on-the-water workouts, and members from the PLYO and E groups rowed together in mixed boats. As all rowers were on the same team, outside of the training intervention (endurance or plyometric), rowing volume and intensity were identical for each member of the study.

### Measurements

All measurements were taken during a single testing session on the Saturdays before and after the 4 weeks of training. Testing occurred over a 90-min period for each participant. All post-testing was performed at least 48 h since the last plyometric or biking session, allowing adequate recovery and avoiding undue influence of the last training session.

Upon arrival, participants had their stature (seca 217, UK), mass (Belfour Inc., WI, USA), and thigh and calf circumferences measured (Gulick tape measure). These measurements were taken by the same member of the research team for pre- and post-testing and were taken half way up the thigh and calf, as has been done in previous plyometric studies [28]. Rowing economy (RE) was assessed with a submaximal 8-min step test on a rowing ergometer (Model D, Concept2, VT, USA), which consisted of four 2-min stages at 90, 120, 150, and 180 W. The Concept2 rowing ergometer is considered to be an accurate rowing ergometer [29] and is the gold standard. Expired gases were collected using a one-way non-rebreathe mouthpiece to determine oxygen consumption (VO_2_), a known measure of energy expenditure (TrueOne 2400, Parvo Medics, Sandy, UT, USA). The accuracy of the O_2_ analyzer is 0.1%, the flowmeter and analyzers were calibrated to less than 1% variance, and typical repeatability (coefficient of variation) in our lab for VO_2_ is ~5%. VO_2_ was obtained during the last 15 s of the 2-min stage, and based upon previous testing and pilot work, we determined participants were able to achieve steady state within the 2-min stages. Rating of perceived exertion (RPE) was measured using the Borgs Scale (RPE 0–10) [30]. Participants completed a 2-min warm-up and cooldown before and after the test, respectively.

After the RE measurements, participants rested for 30 min and then performed a maximal 500-m TT on the rowing ergometer. All participants were familiar with performing maximal 500-m trials as these were performed frequently as part of their normal training and/or performance assessments, prior to enrollment in the study, but none were performed during the intervention period. Stroke rate was not capped for the participants. Time, stroke rate, and average watts were measured during the TT. A 2-min warm-up and cooldown was completed before and after the TT, with participants asked to rest for another 30 min following the TT. Peak rowing power (RP) was then measured over three maximal trials of 15 s on the rowing ergometer. The recorded value for peak power was the highest wattage observed during the 15-s period. For both the RE and TT, the participants were instructed that they could self-select the ergometer resistances, which had to be the same for both the pre- and post-testing. However, as each rower was on the same team, they selected the same ergometer resistances (control 4.4 ± 0.2 vs. plyometrics 4.5 ± 0.0, arbitrary units, *p* > 0.05) which are also in accordance with US National Team testing guidelines. For the RP test, the resistance was set to the highest value (10) on the rowing ergometer.

### Statistical Analysis

All statistics were performed using commercially available software (SPSS v. 23, Armonk, NY). Pre-planned *t* test comparisons were used to determine significance at baseline, in changes within the PLYO and E groups, and at post-test for stature, mass, circumferences, TT performance, and RP. In the case of violating normality, a non-parametric alternative method was employed. In addition, the magnitude of the change from pre-training to post-training was also determined using standardized differences in means (i.e., effect size, ES, Cohen’s *d*) [31, 32]. Threshold values were established as *small* (0.2), *moderate* (0.6), *large* (1.2), and *very large* (2.0). A mixed-model analysis of variance (ANOVA) was used to determine differences in RE. Linear regression analysis was used to determine the slope of the line for VO_2_ across work rate and the intercept for each individual. The level of significance was established at *p* ≤ 0.05. To understand the potential relationships between rowing power, rowing economy, and performance, Pearson correlation coefficients were calculated. Data are reported as means ± standard deviation.

## Results

### Participant Characteristics

During the training period, one subject from the PLYO and one subject from the E group dropped out due to injuries, which were incurred during their on-the-water rowing training and were unrelated to the intervention. Acceptable adherence to the training program was set at 85%, and all remaining participants achieved this or higher. There were no differences in age, stature, mass, or thigh and calf circumferences between or within the groups at baseline prior to training (*p* > 0.05) (Table 1). There no were significant changes in any of the anthropometric measures within either group (*p* > 0.05); as such, at post-testing, there were no differences between the groups for the stature, mass, and thigh and calf circumferences (*p* > 0.05).

### Rowing Economy

**Fig. 1:**
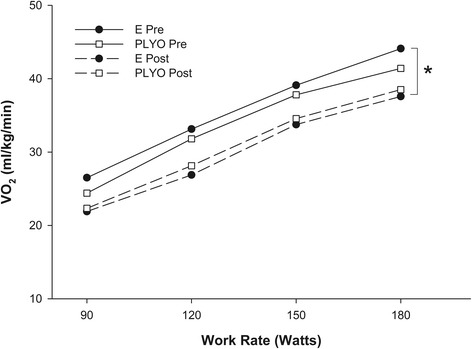
Rowing economy of the endurance (*E*) and experimental (*PLYO*) groups recorded during an 8-min steady-state rowing step test (2-min stages at 90, 120, 150, and 190 W), before (*n* = 9/group) and after 4 weeks of plyometric training (*n* = 8/group). * indicates significant main effect of time (*pre* vs. *post*). Data are presented as means and error bars were omitted for visual clarity

**Fig. 2:**
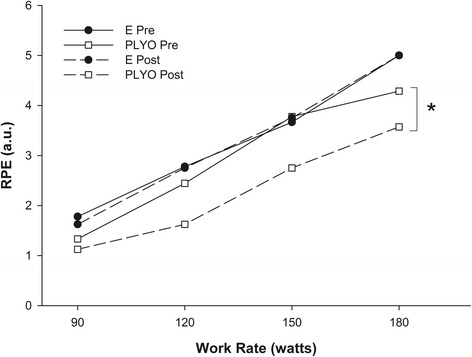
Rating of perceived exertion (*RPE*) during the rowing economy test in the endurance (*E*) and experimental (*PLYO*) groups before (*n* = 9/group) and after 4 weeks of plyometric training (*n* = 8/group). * indicates significant main effect of time (*pre* vs. *post*). Data are presented as means and error bars were omitted for visual clarity

Rowing economy (Fig. 1) did not differ significantly between PLYO and E at all of the work rates before training. In response to training, the analysis of variance indicated no significant interaction effects for VO_2_ values between the two groups at all of the work rates (90, 120, 150, 180 W, Fig. 1, *p* > 0.05) but a main effect for time was found to be significant (*p* < 0.05) indicating a generally lower VO_2_ or energy cost from pre- to post-intervention. Additionally, linear regression analysis indicated no group differences in the slope (VO_2_/watt relationship) at baseline, in response to training, or at post-intervention in either group (PLYO: *p* = 0.17, *p* = 0.18; E: *p* = 0.2, *p* = 0.18). RPE was also taken in the last minute of each stage, and there was a significant reduction in RPE following training in the PLYO group after training (*p* = 0.05), but not for the CON group (Fig. 2).

### 500-m Time Trial Performance

**Fig. 3:**
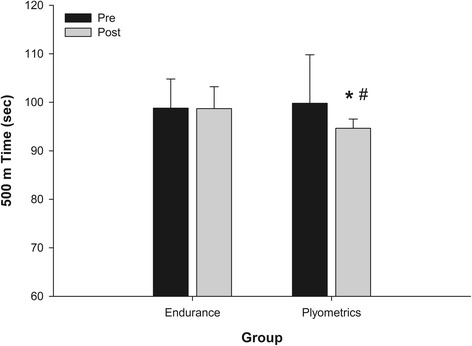
The 500-m rowing ergometer time trial performance of the experimental (*PLYO*) and endurance (*E*) groups recorded before (*n* = 9/group) and after 4 weeks of plyometric training (*n* = 8/group). Data are expressed as means ± SD, * indicates significance within group compared to *pre* (*p* ≤ 0.05)

There was no difference in the 500-m times between the groups before training (*p* = 0.32). TT performance did not change for E after training (*p* = 0.83, ES = −0.08); however, the TT performance was significantly improved in PLYO (Fig. 3, *p* < 0.05, ES = −0.91). Furthermore, difference in TT performance was statistically different between the groups at post-testing. The average power output during the 500-m TT was not different between groups at baseline (363 ± 54 W vs. 372 ± 75 W, E vs. PLYO, respectively, *p* = 0.77), but PLYO significantly increased power output, and thus, the groups were significantly different post-training (368 ± 46 W vs. 414 ± 24 W, E vs. PLYO, respectively, *p* = 0.02, ES = −1.89). Finally, there were no group differences in the average stroke rate during the 500-m TT, at baseline (E 37 ± 4 strokes/min vs. PLYO 38 ± 3 strokes/min, *p* = 0.52), in response to training, or post-training (E 38 ± 4 strokes/min vs. PLYO 39 ± 5 strokes/min, *p* = 0.52).

### Peak Rowing Power

**Fig. 4:**
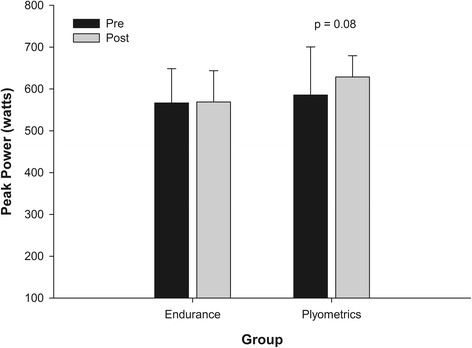
Peak rowing power observed during three trials of 15-s maximal rowing ergometer tests in the experimental (*PLYO*) and endurance (*E*) groups, performed before (*n* = 9/group) and after 4 weeks of plyometric training (*n* = 8/group). Data are expressed as means ± SD

There was no difference in RP between the groups before training (*p* = 0.69, ES = −0.07). After training, there was no change in RP within PLYO or E (Fig. 4; *p* = 0.18, ES = 0.14, *p* = 0.12, ES = 0.11). Although, there was a trend for a difference in RP between the groups following training (*p* = 0.08, ES = 0.66), with a tendency for greater power output in the PLYO group. RP was negatively correlated to VO_2_ (energy expenditure) during work rates 90–180 W before training (*r* = −0.549, −0.879, −0.745, −0.736, *p* = 0.34, *p* = 0.0, *p* = 0.001, *p* = 0.002, for each work rate, respectively). In addition, post-training RP was negatively correlated to the VO_2_/watt slope during the RE test (*r* = −0.576, *p* = 0.039). Finally, peak rowing power was negatively correlated with 500-m time performance before (*r* = −0.92, *p* = 0.0) and after training (*r* = −0.78, *p* = 0.0). Finally, the peak power responses were also highly reliable, and exploration of the three baseline trials indicated a significant reliability coefficient (Cronbach’s *α* = 0.99, *p* = 0.00).

## Discussion

In the current study, we sought to determine if 4 weeks of plyometric training would increase rowing economy, peak power, or performance in experienced youth rowers when combined with on-the-water training. After the 4-week intervention, rowing economy for both groups was significantly improved, but no group differences were found. Peak rowing power was moderately changed as there was a tendency for difference between groups post-training with higher peak power in the plyometric group in response to this relatively short intervention. However, the main finding of this study is that the rowers who performed plyometric training significantly improved their 500-m TT, while this did not change for the endurance group. This study provides the first instance in which plyometric training significantly improved rowing sprint performance.

### Plyometric Training and Rowing Economy

In the current study, rowing economy across work rate was significantly improved in both groups (Fig. 1), possibly indicating that plyometric and aerobic training had a similar effect on rowing economy. Previously, running economy has been shown to improve after plyometric training [21, 24, 28], and the reason for this improvement has been suggested to be increased musculotendinous stiffness (MTS) in the legs of runners [24]. A high MTS increases an individual’s ability to absorb, harness, and recoil force during the stretch-shortening cycle, such as in running [33]. An increase in MTS is thus beneficial for a sport such as running, which involves an eccentric contraction when the runner’s foot makes contact with the ground, where after plyometric training, the increased MTS results in more stored energy in the series elastic elements and reduces eccentric muscle demand. However, the beginning of the rowing stroke (known as the catch), is an anticipated motion in which the athlete is actually pulling themselves towards their feet, followed by a rapid press of the legs to apply power [34]. As there is not so much of a reactive movement, or ground reaction force in rowing when compared with running, increased MTS is likely less beneficial in rowing. Although, plyometric training has also been shown to increase muscle activation [20] and muscle force [35] and decrease ground reaction times [36] suggesting plyometrics might alter the work to rest ratio of rowing, through improved drive speed, allowing more time of the stroke cycle to be spent in recovery for a given stroke rate. Such a change, perhaps independent of economy, might correspond to improved boat speed, allowing for greater maintenance of the forward propulsion or “run” of the rowing shell in the water from each stroke. It is worth noting that these hypotheses warrant further investigation to confirm if this is, in fact, the case.

### Plyometric Training and Rowing Power

Plyometric training has been suggested to improve power [37] in adults and in youth [38]. Results from the current study indicate that plyometric training in this athlete cohort (youth rowers), for this length of time (4 weeks), may elicit a moderate increase in rowing power (Fig. 4). This is in agreement with previous research as plyometrics are typically used to improve power [2, 11, 12]. It has been suggested by rowing biomechanics experts that elasticity stored in the tendons of rowers could be beneficial or initiating the drive sequence of the stroke [34]. The fact that the moderate improvement in rowing power did not reach statistical significance may be attributed to a number of potential factors. The training period was relatively short compared to other studies in the field of plyometrics, so with further training, there could have been a greater and/or significant change in rowing power. The sample size was also relatively small, possibly lacking the statistical power necessary to observe an increase in rowing power. Changes in RP also varied within the groups, suggesting that some individuals may have been better at performing the 15-s peak power ergometer test or possess the innate physiological traits or training acumen to achieve high rowing powers. Also, the motor coordination trained using plyometrics (jumping) is vastly different to the patterns used during the maximal peak power test, with a high rowing resistance. Furthermore, muscle coordination could have differed greatly between individuals due to the athletes being at varying stages of athletic maturity. However, it should be noted that with a greater sample size, and/or a longer training period, a significant difference in peak rowing power could be observed.

The peak rowing power test is a valuable test and predictor of rowing performance, as suggested by Ingham et al. [4]. However, the athletes whom Ingham et al. used were all elite rowers (current or former World Championship Finalists), who have a significantly higher athletic maturity and training history [4] when compared with the athletes in the current study. Therefore, the younger age and relatively lower training history (i.e., coordination and technical mastery) of the athletes utilized in the current study could explain the moderate improvement in rowing power. Specifically, these athletes may not have completed the physical and technical development to truly achieve maximum power, thus perhaps underestimating the possible changes with the training. However, we did find a significant inverse correlation between rowing power and rowing economy, that is, a greater rowing power was associated with lower energy expenditure for a given power output. This is suggestive that rowers with greater power may be more technically masterful and thus expend less energy during rowing.

### Plyometric Training and Rowing Sprint Performance

Plyometric training has been shown to improve short running sprint performance [39], and results from the current study indicate that plyometric training is able to improve rowing sprint performance (Fig. 3). These findings are in contrast to a study performed by Kramer et al. in [3], in which 20 min of simple double leg plyometric exercises were added at the end of the strength training sessions completed by female collegiate rowers. Their rowing measures were a 2.5-km ergometer test, and the distance in meters achieved in 90 s. As a comparison with the current investigation, the 90-s rowing test is very similar to the 500-m TT. The average time for the plyometric group to perform the 500-m TT after training was ~95 s. Kramer et al. found no changes in the 90-s test after 9 weeks of training, whereas we observed a significant improvement in 500-m performance [3]. There are several potential reasons for this discrepancy.

The plyometric exercises used in the earlier study of Kramer et al. [3] were more simplistic than in the current investigation (e.g., no rotation or multiple jumps or upper body involvement). Indeed, the authors did indicate that the exercises they selected may not have been the best for improving rowing performance [3] and were performed following strength training, likely in a semi-fatigued state, suboptimal conditions for plyometric training. Additionally, in comparison to the study by Kramer et al., the current study contained more foot contacts (Table 2.) and focused on plyometric training versus steady-state training, matched for training volume (time) between groups. Kramer et al. did not match training volume [3]. To avoid this, training times were matched for volume of the dry-land training in the present investigation. All on the water rowing training was also matched as all participants were members of the same team. Finally, Kramer et al. [3] recruited collegiate female rowers who were older than the high school male participants in the current study (mean age of 21 compared with a mean age of 16 years). As such, when prescribing plyometric training, it is important to consider factors such as performing these exercises in a non-fatigued state, foot contacts, and load in order to optimize any potential performance improvements.

Nonetheless, the current investigation was able to demonstrate that plyometric training improved 500-m rowing performance (Fig. 3). However, it remains to be seen if plyometric training is capable of improving rowing performance of a more gold standard distance, such as 2- or 6-km TT. As peak power was only moderately increased for the plyometric group, but rowing economy did not differ between pre- and post-training, the improvement in rowing performance could be due to increased rowing power but also to other mechanisms not measured in the current study (e.g., muscle coordination, activation, and rate of force development). Alternatively, the rating of perceived exertion, while not different prior to the intervention, was significantly reduced post-training in the PLYO group only (Fig. 2), which suggests a possible shift in maximal work rate, or the plyometric training may have increased the tolerance for work, thereby reducing perceived effort, and either phenomena might have contributed to the increased performance.

### Experimental Considerations

The current study is not provided without limitation or further consideration. While the 500-m TT was shown to improve as a result of the plyometric training, the training program (three 30-min sessions/week) might be considered time consuming for coaches that are usually working under stringent time constraints. Although, the results from the current study indicate that plyometric training does not impair rowing economy; thus, if on-the-water or ergometer training is not available (e.g., weather or time constraints), plyometric training might serve as an effective substitute or adjunct.

As the purpose of the study was to highlight the effects of plyometric training, we cannot ascertain whether inclusion of strength training or strength training alone would provide additional or superior benefit. Plyometrics are often performed in conjunction with weight training (also known as complex training) and have been shown to cause greater improvements in power than either modality alone [40]. Whether or not complex training is beneficial for rowing performance has yet to be evaluated.

The 500-m TT measure used here is not the gold standard rowing performance test used by rowing coaches. The 2-km ergometer time trial is the most common rowing performance measure [1]. This test not only measures rowing ability, fitness, and technique but also mental fortitude. For this last reason, the 2-km test was not used to evaluate rowing performance of the young athletes in the current study. In young rowers, 2-km performances can vary greatly due to it being mentally challenging so the much shorter 500-m test was used to provide a more objective measure of whether the plyometric program improved rowing performance. Although it is worth mentioning that the reliability of the 500-m TT was not tested, the athletes were very accustomed to the test distance as it is included in training and assessment. Additionally, the athletes were not periodized for the 2-km test, which typically occurs in the spring season. As the 2-km test was not performed in the current study, further research is needed to definitively demonstrate whether improvements in 500-m performance due to plyometrics can be translated into the full 2-km test. The results of the current study can only be applied to youth males, and it remains to be seen if such training responses would be seen in older and/or more developed athletes. Although the 500-m test is not a gold standard measure for rowing, the fact that the athletes were able to significantly improve performance with plyometric training is important. First, because the athletes were able to improve their rowing sprint performance during a time in which no rowing sprint training was being undertaken, this might be a way to preserve anaerobic performance without detriment to aerobic performance (i.e., rowing economy). Second, coaches can also use the 500-m performance to estimate the 2000-m performance, as some coaches consider the wattage produced over 500 m to be 138% of 2000-m watts. Finally, more detailed exploration of the possible changes in muscle will provide greater insight into the training responses to plyometrics in rowers (e.g., hop test and vertical jump).

## Conclusions

The purpose of this study was to determine whether plyometrics, a form of power training already used by rowing coaches, could improve rowing performance and the physiological measures of rowing economy and rowing power. Plyometrics significantly improved the 500-m TT rowing performance and moderately improved peak rowing power, but did not improve rowing economy, suggesting that improved rowing power and other factors could be responsible for the observed increased performance. These results suggest that plyometrics are able to improve rowing performance in young rowers and possibly in older/elite rowers and that rowing coaches should continue using this form of training. Plyometrics can be performed by coaches in conjunction with other methods of strength training or as a warm-up before beginning rowing-specific training. However, further research is needed to determine whether plyometrics can improve 2-km rowing performance and performance of other populations (i.e., elite rowers).
